# Determination of Patterson group symmetry from sparse multi-crystal data sets in the presence of an indexing ambiguity

**DOI:** 10.1107/S2059798318002978

**Published:** 2018-04-24

**Authors:** Richard J. Gildea, Graeme Winter

**Affiliations:** a Diamond Light Source Ltd, Diamond House, Harwell Science and Innovation Campus, Didcot OX11 0DE, England

**Keywords:** Patterson group symmetry, partial data sets, indexing ambiguity

## Abstract

Combining X-ray diffraction data from multiple samples requires determination of the symmetry and resolution of any indexing ambiguity. For the partial data sets typical of *in situ* room-temperature experiments, determination of the correct symmetry is often not straightforward. The potential for indexing ambiguity in polar space groups is also an issue, although methods to resolve this are available if the true symmetry is known. Here, a method is presented to simultaneously resolve the determination of the Patterson symmetry and the indexing ambiguity for partial data sets.

## Abstract 2

A method is presented to simultaneously resolve the crystal symmetry and indexing ambiguity from sparse data sets.

## 1. Introduction   

The recording of an X-ray diffraction data set implies the presence of a crystal lattice with, at the very least, triclinic symmetry. If a relatively complete data set has been recorded from a single crystal, determination of the Patterson symmetry (*i.e.* the symmetry in the diffracted intensities) is relatively straightforward (Evans, 2006 ▸); however, this is more challenging for the partial data sets typical of *in situ* experiments, where diffraction data are collected at room temperature. Further complicating matters is the potential for indexing ambiguity in polar space groups, although methods to resolve this are available if the true symmetry is known (Brehm & Diederichs, 2014 ▸). Determination of the correct Patterson group is a necessary precondition for the correct scaling of X-ray diffraction intensities. The correct group must be compatible with both the observed crystal lattice and the symmetry in the measured intensities. For substantial data sets the unit cell may be accurately determined and the presence or absence of symmetry operators tested within the single set of observations. For sparse data sets this becomes unreliable within one set, and data sets must be combined before analysis. This, however, depends on correctly matching the data sets to ensure that a consistent setting is used, which in turn requires that the symmetry is known.

The correct crystal symmetry must form a subgroup of the crystal lattice symmetry, although in most cases these are identical. If they are not identical one or more ‘twinning operations’ exist which map the true symmetry to internally consistent but mutually incompatible cosets within the lattice symmetry group. In contrast to the conventional problem of indexing ambiguity in polar space groups, for sparse data sets accidental ambiguity is more likely, as the uncertainties on unit-cell constants are greater.

Since the symmetry is unknown at the point of integration of the measurements, it may be appropriate to process the data with a triclinic model and later refine the unit-cell parameters once the symmetry has been determined. This may, however, give rise to up to 24-fold ambiguity if *a* ≃ *b* ≃ *c* and α ≃ β ≃ γ ≃ 90°, in addition to the need to determine the symmetry. Here, we present a method building on that of Brehm & Diederichs (2014 ▸) to simultaneously resolve the determination of the Patterson symmetry and the indexing ambiguity for partial data sets. The approach also addresses cases of accidental unit-cell symmetry, *i.e.* lattice pseudo­symmetry such as a monoclinic cell with β ≃ 90°.

Brehm & Diederichs (2014 ▸) introduced a method for resolving the indexing ambiguity from sparse data sets, and a number of implementations of the method, or related approaches, have since been introduced (Gildea *et al.*, 2014 ▸; Kabsch, 2014 ▸; Ginn *et al.*, 2015 ▸; White *et al.*, 2016 ▸). Their method is a form of the dimensionality-reduction technique known as multidimensional scaling (MDS). The method uses as input the *n* × *n* matrix of pairwise inter-data-set correlation coefficients, where *n* is the number of data sets, and outputs a vector, **x**, of *n* points in *k*-dimensional space, where *k* is generally small (*e.g.* 2 for the case of a twofold indexing ambiguity). In the method presented by Brehm & Diederichs (2014 ▸) each data set is used once in its original setting, and thus is represented by a single point in the vector **x**. They also propose a potential modification of the procedure to include each data set in both its original setting and each of the alternative indexing choices. Here, we present an extension of the methods of Brehm & Diederichs (2014 ▸) and Diederichs (2017 ▸) to all possible symmetry operations of a given lattice group, allowing simultaneous determination of the Patterson group and resolution of any indexing ambiguity.

## 2. Methods   

### 2.1. Dimensionality reduction   

The maximum possible lattice symmetry compatible with the averaged unit cell is determined using algorithms based on ideas by Le Page (1982 ▸) and Lebedev *et al.* (2006 ▸), and implemented in *cctbx* (Grosse-Kunstleve *et al.*, 2004 ▸; Sauter *et al.*, 2006 ▸). Subsequently, a list of all permissible symmetry operations is compiled. The Pearson’s correlation coefficient between data sets *i* and *j*, after application of the *k*th and *l*th symmetry operators, respectively, is defined according to
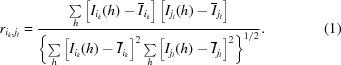
The matrix of correlation coefficients is thus a real symmetric matrix, of size (*n* × *m*)^2^, where *n* is the number of data sets and *m* is the number of symmetry operations in the lattice group.

Following Brehm & Diederichs (2014 ▸), we represent data sets as coordinates, **x**, in a multi-dimensional space; however, in this method each data set appears as *n* × *m* coordinates in an *m*-dimensional space. In the case of pseudo-symmetry, where the true symmetry is *P*1, use of an *m*-dimensional space is necessary to allow the presence of up to *m* orthogonal **x**_*i*_ clusters, where the orthogonality between clusters corresponds to a correlation coefficient 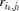 close to zero.

We then use a modification of algorithm (2) of Brehm & Diederichs (2014 ▸) to iteratively minimize the function
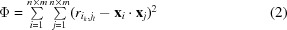
using the L-BFGS minimization algorithm (Liu & Nocedal, 1989 ▸). As in Brehm & Diederichs (2014 ▸), starting coordinates **x** are chosen randomly in the range 0–1.

### 2.2. Principal component analysis   

The procedure outlined above in §[Sec sec2.1]2.1 is performed in an *m*-dimensional space, where *m* is equal to the number of symmetry operators in the lattice group. We anticipate that the points resulting from the minimization above will form a certain number of clusters, given by the ratio of the number of symmetry operators in the lattice group to the number of symmetry operators in the true Patterson group, *i.e.* the number of potential ‘twinning’ operators. Unless the Patterson group is *P*1, the clusters can be represented in a lower dimensional space that is oriented arbitrarily in the higher dimensional space used for the minimization. Principal component analysis (PCA; Pearson, 1901 ▸) may be used to reduce the dimensionality of the resulting clusters of coordinates, which greatly simplifies both the visualization and the further analysis of the clusters. Prior to this analysis, we assume that the true Patterson group, and hence the number of potential twinning operators, are unknown. However, principal component analysis can give an estimate of the relative ratio of the variance of the data that is explained by each principal component, thus giving an indication of the likely number of clusters.

### 2.3. Symmetry discovery   

If the symmetry operator *S_k_* ^−1^ *S_l_* is a member of the true Patterson group, then we would expect the coordinates 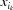 and 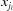 to be part of the same cluster, as the corresponding element of the matrix of correlations, 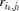, should be close to 1. In contrast, if *S_k_* ^−1^ *S_l_* is not a member of the true Patterson group, and thus a potential twinning operator, then we would expect the coordinates 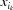 and 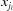 to appear in separate clusters, with a correspondingly lower value of 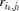.

From analysis of a single cluster, it is possible to identify the Patterson group from the combination of all unique symmetry operators *S_k_* ^−1^ *S_l_* corresponding to pairs of coordinates 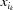 and 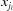. If a potential indexing ambiguity is identified, this can be resolved as follows. If the symmetry operator *S_k_* that corresponds to the coordinate 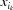 belongs to the Patterson group determined above, then data set *i* does not need re­indexing. If, however, the symmetry operator *S_k_* does not belong to the Patterson group, then *S_k_* is a twinning operator that can be used to reindex data set *i*. Analysis of any further clusters should yield identical results.

The reindexing operator determined using the above procedure will be one from a coset of equivalent reindexing operators. This can be mapped to a unique coset representative using left coset decomposition of the lattice group with respect to the proposed Patterson group (Flack, 1987 ▸).

## 3. Results   

### 3.1. Example 1: simulated microfocus data   

Diffraction patterns for 100 partial data sets were generated by James Holton (Holton, 2015 ▸) from the PDB model of titin (PDB entry 1g1c; Mayans *et al.*, 2001 ▸) as an explicit challenge to the community of macromolecular crystallography software developers. The space group of the generated data sets is *P*2_1_2_1_2_1_, as in the published structure; however, the unit cell has been modified slightly such that *b* = *c*, thus creating a non-obvious pseudo-merohedral indexing ambiguity which must be resolved before merging multiple data sets. The data are intended to be a realistic simulation of the radiation damage to a lysozyme-sized protein forming ∼5 µm crystals shot with a 6 µm beam.

The first three images of each data set were processed with *DIALS* (Winter *et al.*, 2018 ▸) *via xia*2 (Winter, 2010 ▸). No prior space-group or unit-cell information was provided, and integration was performed in *P*1.

The resulting 100 integrated data sets were analysed using the algorithms outlined in §[Sec sec2]2. A resolution cutoff of 3 Å was used; however, the results were not sensitive to the choice of resolution cutoff.

The 100 data sets had a median unit cell of *a* = 38.31 ± 0.03, *b* = 79.11 ± 0.05, *c* = 79.12 ± 0.07 Å, α = 89.99 ± 0.02, β = 89.99 ± 0.03, γ = 90.00 ± 0.01°. The maximum possible lattice symmetry was determined to be *P*422 (space group No. 89), comprising eight symmetry operations.

**Figure 1:**
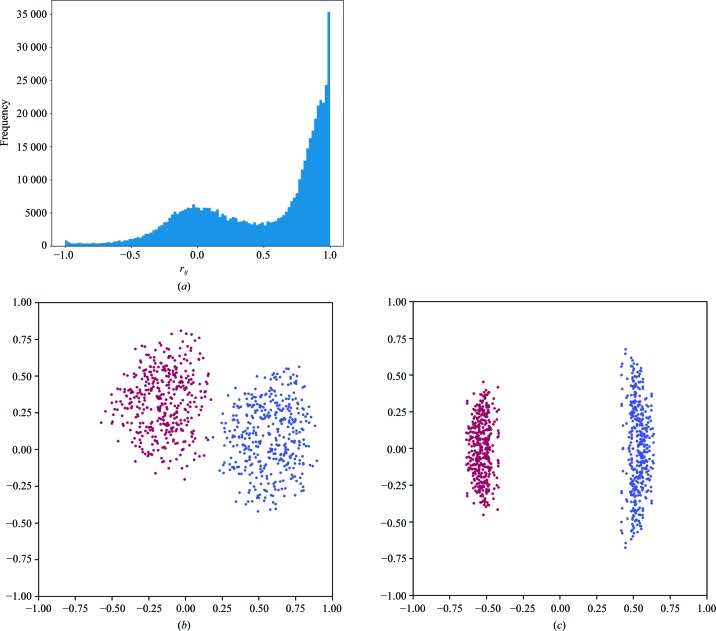
The application of the algorithms in §[Sec sec2]2 to simulated microfocus data sets as described in §[Sec sec3.1]3.1. A histogram of the 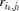 values is shown in (*a*). The points **x** determined by the procedure are shown projected onto the first two dimensions before (*b*) and after (*c*) principal component analysis. Points are coloured according to the assigned indexing mode.

A bimodal distribution of 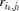 values can be seen in Fig. 1 ▸(*a*), which suggests the presence of an indexing ambiguity. Fig. 1 ▸(*b*) shows the resulting coordinates, **x**, projected onto the *xy* axes, and Fig. 1 ▸(*c*) shows the same coordinates projected onto the first two directions found by principal component analysis. The first direction identified by PCA accounts for 48% of the variance of the data, compared with only 11% for the second direction, and Fig. 1 ▸(*c*) shows that the points are clearly separated into two clusters, reflecting the two possible indexing choices. Two clusters were identified, each containing 400 points, corresponding to four points per data set. Analysis of each cluster according to §[Sec sec2.3]2.3 correctly identified the Patterson group as *P*222.

### 3.2. Example 2: *in situ* membrane-protein data set   

Previously published *in situ* data (Axford *et al.*, 2015 ▸) from an integral membrane protein, *Haemophilus influenzae* TehA (HiTehA), were reprocessed using *DIALS* *via* *xia*2. Processing was attempted on 72 wedges of data consisting of 30–50 images of 0.2° rotation, each wedge therefore consisting of 6–10° of data. No prior space-group or unit-cell information was provided, and integration was performed in *P*1 with the reduced unit cell. Two data sets failed in indexing, leaving 70 data sets which were subsequently analysed according to the algorithms described above.

The 70 data sets had a median unit cell of *a* = 72.58 ± 0.36, *b* = 72.74 ± 0.29, *c* = 72.79 ± 0.23 Å, α = 85.16 ± 0.08, β = 85.19 ± 0.09, γ = 85.29 ± 0.17°. The maximum possible lattice symmetry was determined to be *R*32:r (space group No. 155), comprising six symmetry operations.

**Figure 2:**
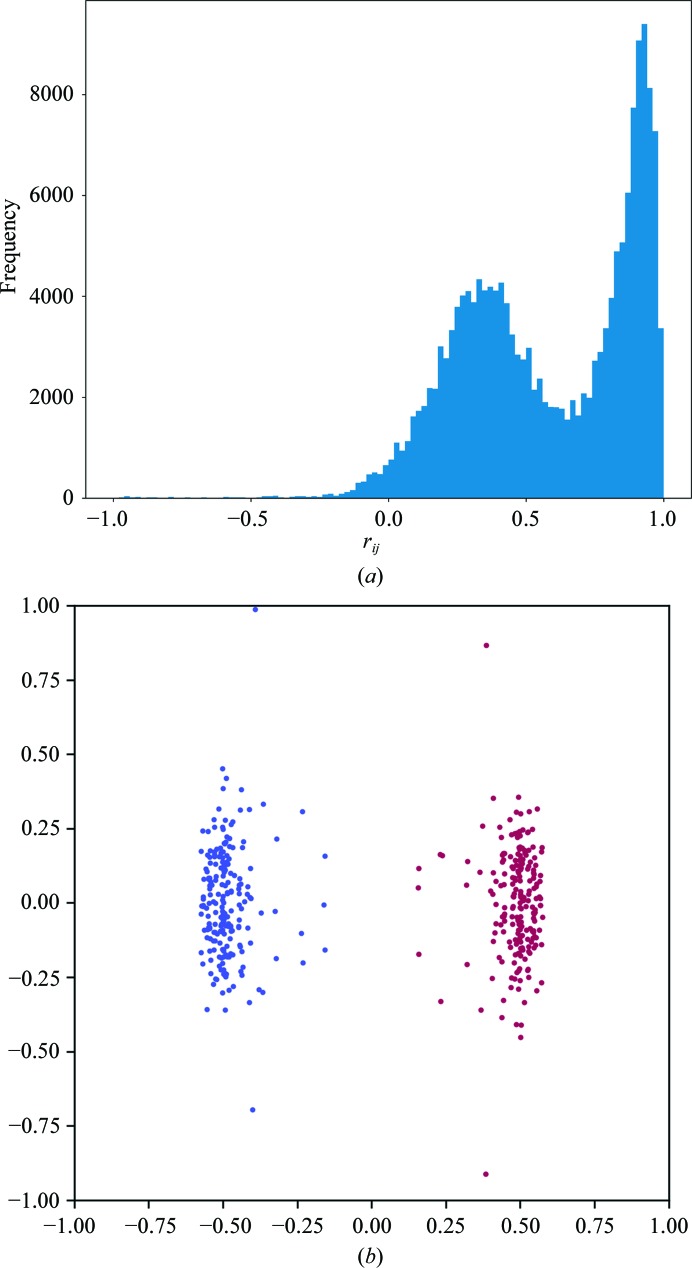
The application of the algorithms in §[Sec sec2]2 to the TehA multi-crystal data as described in §[Sec sec3.2]3.2. A histogram of the 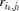 values is shown in (*a*). The points **x** determined by the procedure are shown projected onto the first two dimensions identified by principal component analysis (*b*). Points are coloured according to the assigned indexing mode.

A bimodal distribution of 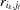 values can be seen in Fig. 2 ▸(*a*), which suggests the presence of an indexing ambiguity. The first direction identified by principal component analysis accounts for 67% of the variance of the data, compared with only 9.6% for the second direction, and visualization of the coordinates after projection onto the first two directions found by principal component analysis in Fig. 2 ▸(*b*) shows that the points are clearly separated into two clusters, indicating the presence of two possible indexing modes. Two clusters were identified, each containing 210 points, corresponding to three points per data set. Analysis of each cluster according to §[Sec sec2.3]2.3 correctly identified the Patterson group as *R*3:h (space group No. 146), which is consistent with the published space group.

### 3.3. Example 3: *in cellulo* micro-crystal room-temperature data set   

Forty 2° wedges of *in cellulo* data from cytoplasmic polyhedrosis virus (CPV) polyhedrin crystals (Axford *et al.*, 2014 ▸) were reprocessed using *DIALS* *via* *xia*2. No prior space-group or unit-cell information was provided, and integration was performed in *P*1 with the reduced unit cell. 28 data sets were successfully indexed and integrated, one of which was rejected after preliminary analysis using the hierarchical unit-cell clustering methods (Zeldin *et al.*, 2015 ▸) available within the *cctbx.xfel* software (Hattne *et al.*, 2014 ▸). The remaining 27 data sets had a median unit cell of *a* = 88.92 ± 0.17, *b* = 89.00 ± 0.14, *c* = 89.04 ± 0.12 Å, α = 109.50 ± 0.08, β = 109.44 ± 0.09, γ = 109.38 ± 0.08°. The maximum possible lattice symmetry was determined to be *I*432 (space group No.211), comprising 24 symmetry operations.

**Figure 3:**
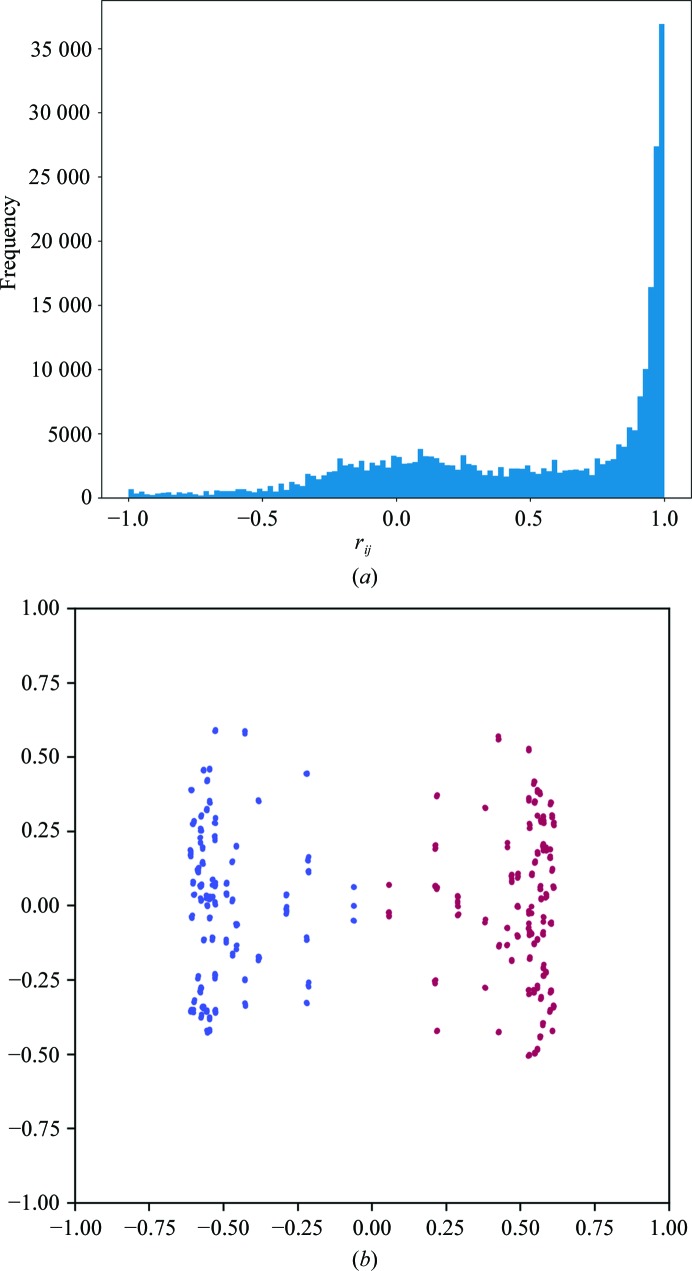
The application of the algorithms in §[Sec sec2]2 to multi-crystal data from CPV polyhedrin as described in §[Sec sec3.3]3.3. A histogram of the 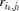 values is shown in (*a*). The points **x** determined by the procedure are shown projected onto the first two dimensions identified by principal component analysis (*b*). Points are coloured according to the assigned indexing mode.

A bimodal distribution of 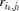 values can be seen in Fig. 3 ▸(*a*), which suggests the presence of an indexing ambiguity. The first direction identified by principal component analysis accounts for 24% of the variance of the data, compared with only 6.2% for the second direction, and visualization of the coordinates after projection onto the first two directions found by principal component analysis in Fig. 3 ▸(*b*) shows a separation of the points into two clusters, indicating the presence of two possible indexing modes. Each of the two clusters identified contained 324 points, corresponding to 12 points per data set. Analysis of each cluster according to §[Sec sec2.3]2.3 correctly identified the Patterson group as *I*23 (space group No. 197), which is consistent with the published space group.

## 4. Discussion   

The results shown in §[Sec sec3]3 demonstrate that it is possible to determine the Patterson group for sparse data sets in the presence of an indexing ambiguity. Three different examples were shown, covering simulated data sets with a pseudo-merohedral indexing ambiguity and previously published *in situ* and *in cellulo* multi-crystal data sets. In all cases, the data were reprocessed in space group *P*1 with no prior assumptions regarding the unit cell or symmetry. Application of the algorithms presented in §[Sec sec2]2 shows a separation of the resulting points into two clusters, representing the two alternative indexing choices. Further analysis of the composition of the clusters was able to correctly identify the correct Patterson group symmetry.

It is noteworthy that while the analysis defined above is predicated on the use of an *m*-dimensional space, where *m* is the number of symmetry operations in the lattice group, in many cases a lower dimensional analysis will give rise to a similar conclusion, particularly where the final number of clusters is small. In the above examples, the analysis was repeated with only two dimensions, resulting in the same conclusions.

Above, we refer to potential twinning operators as the sources of potential indexing ambiguity. The presence of partial twinning would have the effect of making the intensities of the alternative indexing possibilities more similar, thus reducing the separation between the peaks in the histogram of 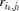 values. This would be expected to reduce the angular separation between the clusters of points, **x**_*i*_, output by the algorithm. As such, we expect our algorithm to be tolerant to the presence of partial twinning when the twin fraction is small, albeit with reduced sensitivity. However, the power of the algorithm to distinguish between indexing modes will rapidly reduce as the twin fraction approaches that for perfect twinning, *i.e.* α = 0.5.

Once any potential symmetry and indexing ambiguities have been identified and resolved, existing methods for the determination of the space group (Evans, 2006 ▸) and clustering of data sets based on unit-cell parameters (Foadi *et al.*, 2013 ▸) and intensities (Giordano *et al.*, 2012 ▸; Diederichs, 2017 ▸; Santoni *et al.*, 2017 ▸) may be used. The algorithms presented here allow data to be integrated in *P*1 with no prior assumptions, with conclusions relating to symmetry derived from the data set as a whole. They therefore make a useful addition to the tools for *in situ* data processing.
